# 

**Published:** 1883-06

**Authors:** 


ORIGINAL COMMUNICATIONS:

Dental Caries.—Dr. W. D. Miller.......... 301
Disease.—Dr. W. H. Atkinson.......... 311
The Proposed Copyright Law.—"Medicus Americanus,".......... 318

SELECTIONS:

The Teeth in Diabetes.......... 321
Swallowing False Teeth.......... 322
Severe Hemorrhage after Tooth Extraction treated by Transfusion... 323
Hot Water as a Beverage.......... 324

EDITORIAL:

The Present Etiological Status.......... 325
A Good Suggestion.......... 328
Explanatory.......... 328
New Department.......... 329
Another Look Ahead.......... 329
The Springfield Meeting.......... 332
A Liberal Offer.......... 333

CURRENT NEWS AND OPINION:

Educational Association.......... 333
The Odontological Society.......... 334
Pennsylvania State Board of Dental Examiners.......... 334
Detroit Dental Society.......... 335
Indiana State Dental Association.......... 335
A Singular Statement.......... 336
The A. M. A.......... 336

BIBLIOGRAPHICAL:

Our Book Table.......... 337

ORIGINAL TRANSLATIONS AND ABSTRACTS:

Foreign Bogus Diploma Mills.......... 340
Propagation of Bacteria.......... 340
Adulterated Foil.......... 341
German Dentistry.......... 342
What a Record.......... 342
An Alveolar Dilator.......... 342
Extraction with Gas.......... 343

POPULAR SCIENCE DEPARTMENT:

Americanitis.......... 344
Poor Teeth the Cause of Many Ills.......... 345
The Speed of Thought.......... 345
Iodoform in Toothache.......... 346
Dust, Mist and Clouds,.......... 347
Pepsin in Sea Sickness.......... 347

